# Attitudes towards advance care planning amongst community-based older people in England

**DOI:** 10.1371/journal.pone.0306810

**Published:** 2024-08-21

**Authors:** Sara Spear, Ed Little, Alan Tapp, Clive Nancarrow, Yvette Morey, Stella Warren, Julia Verne

**Affiliations:** 1 Faculty of Business and Law, St Mary’s University, London, England; 2 Bristol Business School, University of the West of England, Bristol, England; 3 OHID, Department for Health and Social Care, London, England; Singapore General Hospital, SINGAPORE

## Abstract

**Background:**

Advance care planning has been advocated as a way for people to have their wishes recorded and respected in relation to types of treatment and place of care. However, uptake in England remains low.

**Aims:**

To examine the views of older, well, adults towards Advance Care Plans (ACPs) and planning for end-of-life care, in order to inform national policy decisions.

**Methods:**

A mixed methods approach was adopted, involving individual and mini-group qualitative interviews (n = 76, ages 45–85), followed by a quantitative survey (n = 2294, age 55+). The quantitative sample was based on quotas in age, gender, region, socio-economic grade, and ethnicity, combined with light weighting to ensure the findings were representative of England.

**Results:**

Knowledge and understanding of advance care planning was low, with only 1% of survey respondents reporting they had completed an ACP for themselves. Common reasons for not putting wishes into writing were not wanting/needing to think about it now, the unpredictability of the future, trusting family/friends to make decisions, and financial resources limiting real choice.

**Conclusion:**

Whilst advance care planning is seen as a good idea in theory by older, well, adults living in the community, there is considerable reticence in practice. This raises questions over the current, national policy position in England, on the importance of written ACPs. We propose that policy should instead focus on encouraging ongoing conversations between individuals and all those (potentially) involved in their care, about what is important to them, and on ensuring there are adequate resources in community networks and health and social care systems, to be responsive to changing needs.

## Introduction

Advance care planning is internationally regarded as an important element of public health policy in palliative care. The notion of advance care planning can be dated back to the introduction of advance directives or ‘living wills’ in the 1970s in the US, and subsequently, Power of Attorneys for Health, and Do Not Attempt Resuscitation (DNR) orders [1]. In the UK, these are known as Lasting Power of Attorneys, and the Advance Decisions to Refuse Treatment (ADRT) process [2]. The shortcomings of these legal approaches though, which tend to be formal, fixed, and transactional, have led to the advance care planning approach, which is more communications focused [1]. Advance care planning aims to increase the autonomy of the individual in treatment and place of care decisions, and it has been suggested that advance care planning achieves better outcomes for individuals, and can also increase the cost effectiveness of end-of-life care [3–5]. Since 2008, End-of-life Care Policy in England has emphasised the value of written Advance Care Plans (ACPs); documents which record patients’ preferences for the future, including treatment options at the end of life, and which can be accessed by all those involved in giving care [6,7].

In many countries, including England, the completion of ACPs is limited though [8,9]. ACPs are regarded as particularly important for some specific groups, such as those with a terminal condition, cancer, or dementia diagnosis [10]. There are similarities between ACPs targeting these groups and the ‘goals-of-care discussions’ and ‘serious illness conversations’ introduced in some hospitals. Goals-of-care discussions focus on patients with chronic life-limiting illnesses, facing crucial treatment decisions [11]. Serious illness conversations use physical deterioration and the need for palliative care as a key prompt [12], and focus on the current and future care needs of patients nearing the end of life [13]. There is, however, a view that ACPs should also be completed by people we will term here as ‘older well’, that is, older people still in reasonable health and living largely independent lives in the community [5,10]. Most of the literature on ACPs focuses, either explicitly or implicitly, on these specific health-limited groups, neglecting the community-based older well [4,14,15]. Given that older, well, people form such a large proportion of the potential market for ACPs, very little is known about the views of the older well towards advance care planning. This paper aims to inform health policy internationally and (in particular) in England, by presenting the results of a mixed methods exploration of the attitudes and behaviour of older, well, people in regard to ACPs.

## Literature review

There is general agreement in the literature regarding the potential benefits of advance care planning. In a review of 113, mostly American, empirical studies, Brinkman-Stoppelenburg et al [4] found that advance care planning tended to reduce the amount of aggressive, painful, life-prolonging interventions, increase the use of palliative care, reduce the frequency of hospitalisation, and increase the use of hospices. Similarly, in the UK, Abel et al [16] and Dixon et al [17] found that ACPs were significantly associated with reduced hospitalisation towards the end-of-life, whilst Orlovic et al. [18] found that the likelihood of a hospital death increased when patients did not have a documented preferred place of death. The study by Abel et al. [16] included data from 969 patients known to one hospice across a two-and-a-half-year period, of which 550 completed advance care planning. Dixon et al. [17] undertook secondary analysis of 22661 responses from the National Survey of Bereaved People 2013, and Orlovic et al. [18] analysed 21231 individual records from an electronic palliative care coordination system. The large samples in these studies give a robust insight into the outcomes of advance care planning. Interest in advance care planning is increasing in Asian countries too, driven by national programmes, such as that in Taiwan and Singapore [19,20], although there remains low awareness of ACPs [21]. An earlier study by Prendergast [22] found though that improved outcomes were associated with improved communication processes between patients and care providers, rather than with increased documentation.

There is considerable evidence that the general population views the idea of advance care planning, in principle, as a valuable activity which everybody should undertake [23–25]. Despite this, completion rates remain low. Even in the United States, where the 1990 Patient Self-Determination Act requires all federally funded medical facilities to check whether patients have an ACP and to make forms available to those who do not, Rao et al [26] found that only 26% of the population had an ACP. In studies of the general population of the UK, The Netherlands, Belgium, and Australia, the corresponding figure ranges between 1.8% and 7% [9,14,24,26]. There is a clear distinction between people in different life-stages; studies of people in hospitals, hospices, and residential care facilities, or those who have been diagnosed with a life-limiting condition, have estimated the take-up of advance care planning to be between 16% and 48% [27], whilst for the community-based older group, it is around 12% [9,28].

The generally poor uptake of advance care planning is attributed to several barriers, most related to planning uncertainty. This includes uncertainty about when to initiate the planning process [23,29], difficulty predicting future care requirements [29,30], and lack of knowledge about how to produce an ACP [15,31]. Brinkman-Stoppelenburg et al [4] argued that there was little evidence of any benefit from formulating an ACP *before* the point at which an individual has a diagnosis of a life limiting condition. Healthcare providers have also reported difficulties in planning, for patients other than those with a terminal illness [8,10,32]. Piers et al [33] found that uncertainty about death as a possible future outcome often prevented engagement with planning. The subjects of their study were aged 70 and over, identified by healthcare professionals on the basis that it would not be surprising if they were to die in the next 6 months. Even among this group, a significant number refused to accept that their death was sufficiently likely or imminent to warrant the development of a care plan. This uncertainty is worse in the case of community-based older people, whose care needs will be less predictable than those of a patient with a specific illness [8]. A reluctance to talk or think about death is also cited as a barrier to advance care planning [10,30].

Considerable attention has been devoted to the development of strategies to overcome these barriers. Important facilitators of advance care planning are trust in healthcare providers and family members [29,30,34], communication [22,30,35], education, procedures, and resources [15,32,34]. There is some evidence that complex interventions involving an ongoing communication process between several different healthcare providers, as well as family or community carers, are the most successful at encouraging ACP completion [8,22,30]. However, several authors have noted the high level of resource needed to implement such behaviour change interventions [8,14,32].

Given the calls for ACPs to be initiated amongst the older well [5], and calls for more research on how this group regards the various facilitators, barriers, and benefits of advance care planning [4,8,14], the research we report on here is timely. Our research aims to help guide future policy through a deeper understanding of the older, well, public’s attitudes and behaviours towards ACPs, including exploring reactions to the generic proposition of ACPs, and personal disposition to plan (or not), and reasons why (not).

## Method

The study was undertaken in two phases, using a mixed methods approach; phase 1) qualitative interviews (individual and mini-group), phase 2) quantitative online survey. Ethical approval for the research was granted by the University of the West of England’s Faculty Research Ethics Committee on December 4^th^ 2018 (Ref: FBL.18.10.014 Tapp). Written informed consent was obtained from all participants. Interview participants were provided with an information sheet and completed a consent form before the start of the interview. Online survey participants were provided with the information at the start of the survey and were required to click to confirm their consent before proceeding to the survey.

### Phase 1 –qualitative

The qualitative phase of research facilitated an in-depth understanding of perceptions and experiences of advance care planning. The first level sampling criteria consisted of residence (in England) and age (45–85). The latter was divided into a primary target group aged 68 (post-retirement) to 85 who fit the ‘community-based older well’ criteria (e.g. reasonably fit and well, without life-limiting diagnoses, and not living in a care or nursing home). A secondary target group of ‘informal influencers’ was also identified, of people aged 45–67 who had an elderly relative. This was raised to 55–67 in the second stage of interviews, as we found that this latter group had greater experience with elderly relatives. We worked with a fieldwork company to recruit participants for the qualitative research, and they used their network of local recruiters within the North West and South West of England to recruit members of the public in accordance with our criteria. These regions were selected in order to gain a breadth of views within England, and also for feasibility within the budget and timescales of the funder. An interlocked quota sampling strategy was used to achieve a balance of male and female participants across the primary and secondary target groups.

Seventy-six participants aged 45–85 took part in the qualitative phase, with thirty individual interviews and 12 ‘friendship’ (people who knew each other prior to the discussion) mini-group interviews, with three or four participants in each. The mix of in-depth and mini-group interviews helped elicit both personal and social perspectives. Data collection was carried out in three iterative stages, with time in between each stage for the interviewers to reflect on and make improvements to the conduct of the interviews, the topic guide, and the interview stimuli. We set a quota of 15 participants for the pilot fieldwork, and 30 participants for the first main stage of fieldwork, to enable this reflection and enhancement. Recruitment then continued in the second main stage of fieldwork until the research team were confident that they had reached data saturation point. Fieldwork took place between 10^th^ December 2018 and 28^th^ June 2019, with the pilot fieldwork undertaken in December- January, and two main stages of fieldwork carried out March-April, and May-June, respectively.

The initial interview guide (S1 File) was developed in response to the research aim, informed by the literature review. A descriptor of an ACP was introduced after exploring existing awareness of and attitudes towards ACPs (S2 File). In the final stage of interviews, a specific example of an ACP was shown to participants as a stimulus for further discussion (S1 File). Interviews were audio-recorded and transcribed verbatim. Transcripts were imported into NVivo12 to facilitate data management and analysis. Qualitative analysis took the form of pattern matching [36], in which a series of propositions were generated from the literature regarding the facilitators of and barriers to the Advanced Care Planning, against which cases in the dataset were compared. The initial deductively derived themes related to the study objectives, focusing on general attitudes to ACP (with codes for positive and negative attitudes) and specific barriers and facilitators that were evident in the extant research (with codes including attitudes to death, family relationships, attitudes towards the medical profession and autonomy and choice in end-of-life care). Since the data collection took place over three stages, the research team inductively added to this in the light of findings from each stage. For example, the theme of attitude towards death was developed into two distinct themes, distinguishing between death itself and the process of dying. Similarly, when analysing attitudes towards the medical professional, it became necessary to code separately for attitudes towards GPs, hospital consultants and those providing nursing care. All themes and codes were discussed and agreed by the research team.

### Phase 2—quantitative

The quantitative phase set out to explore the qualitative findings with a larger sample of the population. A cross sectional online survey was conducted with adults aged 55 or older in England, with a stratified random sample drawn from the 500,000+ YouGov UK online access panellists. YouGov are a Member of the British Polling Council, and their online panels consist of members of the public who have agreed to answer questionnaires online on a variety of topics for points contributing to prizes. The 55 plus age criteria was determined based on the insights from the qualitative phase, as this covered older adults who were the target group for writing their own ACPs, as well as adults who would have experience of elderly relatives in this target group. Primary sampling strata were age, gender, socio-economic status, and region. The main fieldwork took place between 1^st^-21^st^ September 2020, with a test of the questionnaire conducted with 104 respondents several days before the main fieldwork. YouGov reported that 53% of those sent an email invite to participate opened the questionnaire and 91% of these went on to complete it in full. This yielded 2,590 respondents. Questionnaire presentation was designed to be user friendly on all devices, with one question per page. YouGov safeguard the anonymity of respondents and their privacy policy is available here. The publication of survey results adheres to the Rules of the British Polling Council [37].

The questionnaire design was informed by the qualitative research and findings. We used the themes from the qualitative findings to determine the topic areas for the survey questions, and we drew upon both the findings and the literature, to determine the pre-set responses to the questions. The final questionnaire (S2 File) included closed questions, such as 5 point (Likert) agree-disagree scales, and open-ended text questions. Where appropriate, items in a list and the order of statements for Likert scales were randomised, to balance out any order effects. Also where appropriate, text boxes were added to pre-identified lists to capture responses not catered for, creating a more satisfying respondent experience and encouraging co-operation. The authors specified cross tabulations, layout of tables, and any cumulative counts of similar respondent sentiments. Whilst using an online panel excludes those without access to technology, it does have key strengths which we deemed important for this research, in particular reducing bias from socially desirable responding, by interviewers not being present. Using an online mode also meant respondents could work at their own pace, which is relevant for older respondents. The sample size was determined based on the desired coverage of demography, level of sampling accuracy required, and planned analysis methods, and a target of 850 respondents in each of three key age groups (55–64; 65–74; 75+) was set. Results were provided by YouGov using their proprietary software (Gryphon). The data in the Findings show effective sample sizes (unweighted sample sizes corrected to take into account the effect of weighting on the margin of error). These are the correct bases for statistical tests and inferences.

Conducting the research in two phases, and using mixed methods, enabled us to triangulate the data from the qualitative interviews and mini-groups with the quantitative survey findings. This strengthened the validity of our findings, and the depth and generalisability of our insights.

## Findings

The profile of participants in the qualitative stage is presented in Table 1. Seventy-six interviews and eight mini-groups were conducted with the primary target group (aged 68–85, ‘community-based older well’), and one interview and four mini-groups were conducted with the secondary target group (aged 45–67, ‘informal influencers’). Respondents all lived in the community (rather than care/nursing homes), and were based across the North West and South West of England.

**Table 1:** Profile of participants in qualitative phase.

Characteristic	Participants *n = 76*
**AGE**	**%**
45–67 (depth interview)	1
45–67 (mini-group)	12
**45–67 (total)**	**13**
68+ (depth interview)	29
68+ (mini-group)	34
**68+ (total)**	**63**
**GENDER**	
Male (depth interview)	14
Male (mini-group)	22
**Male (total)**	**36**
Female (depth interview)	16
Female (mini-group)	24
**Female (total)**	**40**
**REGION**	
North West (depth interview)	8
North West (mini-group)	24
**North West (total)**	**32**
South West (depth interview)	22
South West (mini-group)	22
**South West (total)**	**44**

The profile of respondents in the quantitative phase is presented in Table 2. The sample was based on quotas in age, gender, region, socio-economic grade, and ethnicity combined with light weighting to ensure the findings are representative of England. Nine out of ten respondents claimed to be in “fair” to “very good health”, thereby representing the ‘older well’.

**Table 2:** Profile of respondents in quantitative phase.

Characteristic	Respondents *Unweighted n = 2590* *Effective n for statistical inference = 2294*
**AGE**	%
55–64	39
65–74	33
75+	28
**GENDER**	
Male	47
Female	53
**REGION**	
North	27
Midlands	30
South (exc. London)	27
London	16
**SOCIO-ECONOMIC GRADE (in ordinal position with AB highest)**	
AB	21
C1	29
C2	23
DE	27
**ETHNICITY**	
White	92
BAME	6
Prefer not to respond	2
**HEALTH IN GENERAL**	
Very good	19
Good	46
Fair	25
Bad	7
Very bad	1

### Awareness of ACPs

In the survey, respondents were shown a list of eleven activities or services potentially relevant to older people, such as making a will and funeral plans (S2 File). Claimed awareness, knowledge, and experience of ACPs were the lowest of all the activities, with 28% of people aware of ACPS (this and other survey data are presented in Table 3). Open ended questions asked to the 4% (110 respondents) who claimed they could “fully explain advance care plans to someone else” resulted in 10 respondents stating they did not know when to do one, 33 suggesting it should be done while capable, and 16 saying or implying “anytime” or now. When the 110 were asked what should be stated in an ACP, 54 respondents gave vague answers, and a further 20 said “don’t know”. Of the other respondents, 25 referred to Do Not Resuscitate (DNR), and 20 to where one would wish to live (home or care home). These free text responses suggest patchy knowledge of advance care planning amongst even the most confident respondents. This echoed the findings from the interviews and mini-groups; participants had not heard the specific term ‘Advance Care Plan’, and were not aware of what an ACP was. They were similarly not aware of the general policy or the expectation that they should complete an ACP.

**Table 3:** Survey data.

Criteria and topic	%
**Adults aged 55+ (n = 2294)–awareness of ACPs**	
Aware of	28
Claim could fully explain to someone else	4
Looked into for either self or someone else	2
Completed for self	1
Helped someone with	2
**Adults aged 55+ (n = 2294)—thinking about and communicating wishes**	** **
I haven’t thought about what my wishes are	54
I know what my wishes are, but I just haven’t told anyone or written them down	17
I have told someone my wishes, but haven’t put these in writing	17
I have put my wishes in writing	7
Not sure/ can’t remember	5
Tell someone my wishes for my care	31
Put my wishes for my care in writing	35
Neither–no intention of doing an ACP	34
**Adults aged 55+ who would tell someone/put their wishes in writing (n = 1523)—whom would involve**	** **
My partner	55
Another relative	54
Solicitor	15
Close friend	12
GP	12
My hospital consultant	5
Care home/nursing home /assisted living manager	2
Other/Don’t know	9
No intention of doing an ACP	3
**Adults aged 55+ who would put their wishes in writing (n = 805)—whom would entrust to keep the plan**	
My partner	52
Another relative	48
Solicitor	32
GP	14
Close friend	12
My hospital consultant	4
Care home/nursing home /assisted living manager	2
Other/Don’t know	8
**Adults aged 55+ who have not put their wishes in writing (n = 2118)—reasons why**	
I don’t want to think about it yet	24
I can’t predict my future health, finances, or who might be able to help me	22
No need as I trust my family/friends to step in	18
I don’t need to think about it yet	16
No need as my family/friends would know my wishes	15
I don’t know how to write an ACP	14
I don’t think that having an ACP will make any difference to the care I receive.	12
I have other more important or pressing issues in my life	11
I don’t want to do one in case my circumstances change	9
Don’t know to whom to give a written ACP	8
Don’t know whom to trust with my ACP	6
An ACP could get ignored	6
Writing an ACP would upset me	4
Forgot to do anything about it	4
Writing an ACP would upset any family/friends that I involved in the process	3
No need as Social Services and the NHS would look after me	3
An ACP could get lost	3
Other/Don’t know/ None of these	21
**Adults aged 55+ who saw alternatives to putting wishes in writing (n = 2118)—% agreeing**	
Be more inclined to simply tell my family/friends rather than produce a written ACP	55
Be more inclined to write a will as something positive to do now	52
Prefer to appoint someone with a Lasting Power of Attorney for my health and welfare	42
Be more inclined to plan my funeral as something positive to do now	29
**Adults aged 55+ (n = 2590)—potential prompts to do an ACP**	
So my family would know what I want in terms of care	41
A serious illness/ condition affecting me	41
It could help me to stay at home rather than go into care	34
Concerned that my family would not know what type of care I would want	24
It could help me get the type of care I want	23
It would help doctors and nurses to make the right decisions for me	21
A serious illness/ condition affecting someone else close to me	20
A GP suggesting it	17
A hospital consultant suggesting it	15
A friend/relative suggesting it	9
A nurse suggesting it	7
A pandemic like Covid 19	5
Learning about it in the media (TV/radio/press/social media)	4
A carer suggesting it	3
Discovering it on the internet	2
Other/ Don’t know / Can’t remember / None of these	28
**Adults aged 55+ with elderly relatives/neighbours/close friends (n = 1625)—raising ACP**	
I feel it is not my place to start the conversation with any of them	49
I would be embarrassed to start the conversation with any of them	12
I think it would be a good idea for me to initiate the conversation with some of them	11
It would be easier to focus on advance care planning for them rather than to do one myself	4
I would need to know more about advance care planning	18
None of these	18

### Attitudes towards ACPs for self

Following the awareness questions, a description of advance care planning was shown, on screen to the survey respondents, and on a printed card to the interview respondents (S1 and S2 Files). Survey respondents were then asked ***“****Have you written an Advance Care Plan for yourself covering these issues or told anyone about your wishes if you were to need care*? *Which one statement below best describes you*?*”* The responses showed that the majority have either not thought about their wishes (54%) or have not communicated them (17%). Only 17% claimed to have informed someone of their wishes, and 7% claimed to have put their wishes in writing (presumably, not in the form of an ACP, given the earlier results shown in Table 3, where only 1% claimed this). Of course, it may be the case that the term ‘Advance Care Plan’ was not fully recognised or understood. Older respondents, females, and those in the highest socio-economic group (AB) were most likely to have communicated their wishes in some way (writing or telling).

Respondents were also asked **“**Would you prefer to tell someone your Advance Care Plan wishes or put them in writing?”. “In writing” marginally edged “telling” someone (35% versus 31%, which is statistically significant at the 95% level). Younger respondents were more inclined to favour “in writing”, as were women. Around one third (34%) stated they had no intention of doing an ACP.

Those stating that they would communicate their wishes either in writing or verbally were asked who they would trust to involve. A partner was selected as the most likely choice overall, particularly in younger age groups and by men. It may that some (male, in particular) partners in the older age group may have died, or that partners of either sex were regarded as less cognitively competent as they got older and so possibly represented an unwise choice. Only 15% cited a solicitor. Using the same list, those who stated a preference for putting their wishes in writing were asked who they would trust to keep the ACP. The choices largely reflect the previous question on involvement, though solicitors become more prominent, particularly amongst the younger age group. Medical professionals were a relatively infrequent choice for both questions.

The qualitative findings offer insight into these choices of whom to involve in advance care planning. GPs were generally not trusted to offer the specific expert advice that respondents typically wanted, nor were relationships with GPs sufficiently strong to warrant their inclusion in the planning process.

*I don’t think your family doctors are like your family doctors from years ago, they knew everything about you… but life’s not the same now is it?* [Female, 85]

Some expressed more trust in medical staff in hospitals. Consultants were generally trusted with life-or-death decisions, and many respondents were content for them to overrule the directives in an ACP:

*I think nothing is written in stone and I think that the doctors should be able to overturn things, particularly in prolonging life.* [Male, 73]

The National Health Service (NHS) in general was viewed very positively. However, it was widely felt that the NHS was too overworked and under-resourced to take on responsibility for the storage and dissemination of ACPs.

*Not the NHS…, I think they’ve got enough on their hands. So to be honest with you, I think it’s better to have somebody just legal, like a solicitor or an accountant.* [Female, 68]

### Barriers to ACPs

The qualitative interviews uncovered a range of reasons why people did *not* write an ACP for themselves. Survey respondents were presented with this list of reasons, and could select as many as applied to them. It was striking that many interview respondents felt that advance care planning was a good idea–but *not yet*:

*…so I wouldn’t fill that in yet, but if I was told I was poorly then I would…you have to be in a mental state to do that because at the moment we think we’re a bit invincible.* [Female 68]

The ‘not yet’ factor was the largest single factor emerging from the survey findings, with 24% choosing “I don’t want to think about it now”, 16% “I don’t need to think about it now” and 11% agreeing that “I have other more important or pressing issues in my life”. This reluctance to think about advance care planning appeared to stem from *fear of deterioration*, rather than fear of death itself. Respondents were particularly unwilling to contemplate their future personal care requirements:

*When you’re ill at home when you’re old, what happens when you go to the toilet? You don’t want your children to wipe your bottom, do you.* [Female 72]

Secondly, the *unpredictability of future life* looked to be off-putting to many, with 22% agreeing “I can’t predict my future health, finances or who might be able to help me” and 9% choosing ‘I don’t want to do one in case my circumstances change’. This was also frequently expressed in the interviews:

*We just don’t know what we will require. We don’t know if we’re going to become physically a problem, mentally a problem, or just age-related mobility. So when you don’t know potentially what the requirements are, you can’t really put it down and plan it.* [Male 70]

A third issue could be described as *faith in others*, *particularly those close to you* with 18% agreeing there was “no need as I trust my family/friends to step in” and 15% saying there was “no need as my family/friends would know my wishes’.

*If you’ve got a good family, they will help, you don’t have to ask them* [Female 80]

Far fewer seemed to have faith in the health and social care services though, with only 3% agreeing there was “no need as Social Services and the NHS would look after me”. Care homes, in particular were viewed with trepidation:

*when they’re in a care home, the staff are constantly changing and nobody takes the time.* [Female 57]

Despite this general lack of faith in health and social services, only 6% believed that an ACP could get ignored. This could be taken as a trigger to write an ACP, but the low uptake discussed previously suggests this was insufficient motivation to bother.

A fourth issue highlighted in the qualitative data was the *reality of lack of choice*. Despite ACPs being promoted as enabling choices, options are often limited for many people. Money was discussed by interviewees as one factor which affected choices:

*…if you’ve got the money you can pick and choose where you go, if you haven’t got the money unfortunately they’ll put you wherever they’ve got a space.* [Male 70]

In most cases, therefore, the potential benefits an ACP did not seem to compensate for the psychological discomfort involved in formulating one.

To probe their views in more depth, survey respondents were asked to what extent they agreed/disagreed with several statements related to advance care planning. Their responses indicated a preference for other activities as an alternative to completing an ACP.

“Telling friends/family your wishes” continued the theme discussed previously, of having faith in others, with 55% of people preferring to do this, rather than write an ACP. This reliance on friends/family could be formalised through a Lasting Power of Attorney for Health and Welfare, which 42% were inclined to do, and which would empower specific people to adopt the role of surrogate decision-maker if their loved one were to lose mental capacity to make decisions. Writing a will or planning a funeral were both selected as other positive actions, enabling an individual to focus on the practical aspects of death where there is less uncertainty, rather than the more uncertain planning for care when dying. This was apparent in the interviews:

*I made a will and I’ve got a funeral plan with the Co-op, they took care of it, and it’s already paid for. (Wife)’s the first one on the will, she gets a lump sum, and the boy and my daughter get a share of what’s left.* [Male 68]

Participants were comfortable talking about wills and funeral arrangements. Those who had made these plans were proud to be able to relieve the burden for, and benefit, those left behind.

### Triggers and timing

The qualitative research indicated potential triggers for completing an ACP, and these were explored further in the quantitative stage. Respondents were asked “*what prompted you or would prompt you to think about an Advance Care Plan for yourself*?” The answer list was based on the qualitative findings, and respondents could select as many as applied to them.

Making family aware of wishes for care, helping one to stay at home rather than go into care, and a serious illness/condition affecting them were the key prompts for individuals to do an ACP. Women were more likely to be persuaded by the idea of getting the care they want or need–perhaps men may be happier to rely on women to look after their care requirements. There was little consensus on who might prompt one to do an ACP (GPs 17%; hospital consultants 15%; a friend/relative 9%; a nurse 7%; a carer 3%). The least likely to be swayed by a GP were the 75+ group and females, though the differences were small. Given the timing of the survey, we explored the influence of Covid-19 on advance care planning. However, “a pandemic like Covid-19” was only seen as a trigger to do an ACP by a small number of people (5%). We probed this further in later questions, and only 15% of people agreed that the Covid-19 pandemic made them think more about their plan for end-of-life care, and only 8% agreed that the pandemic had got them talking to others about end-of-life care. As this was the position at the height of the pandemic, Covid-19, or a similar threat, is likely to be even less of an influencing factor now or in the future.

In the interviews, the diagnosis of a serious illness was discussed as the trigger for writing an ACP:

*If I had an illness, I would have to have them all (family) here, and I would say like this is what I want, it would be all written down.* [Female 68]

However, in reality, even some of those who had recently suffered from an acute illness had not seen this as an imperative to produce a plan:

*I’ll wait for a serious illness, but saying that I’ve had cancer on the throat, I’ve had cancer in my stomach, and I’ve had um cancer in my kidney (…) I’d wait for the trigger if there’s a serious illness.* [Male, 70]

This relates back to the small number of people who have actually completed an ACP, and indicates the mental barriers that individuals have to overcome to decide that their health is poor enough to warrant completing an ACP.

### Advance care planning for others

Seven out of ten respondents had elderly relatives, neighbours, or close friends, and this group were asked about their feelings on raising the topic of advance care planning for any of them to consider. Respondents could choose more than one statement as they may have felt differently about the various older people in their lives, but the great majority chose just one statement. Only a few people (11%) thought raising the topic was a good idea. The majority give various reasons not to, 49% saying it would not be their place, 12% that it would be embarrassing, and 18% saying they needed to know more about it.

In most cases, therefore, the potential benefits an ACP did not seem to compensate for the psychological discomfort involved in discussing one. The implications of these findings are discussed next.

## Discussion

In common with other studies [10,23–25] we found that respondents reacted well to *the idea* of ACPs. However, this generally did not lead to a personal disposition to complete an ACP. There were numerous reasons given for not ‘acting now’, with many linked to the uncertainties of future health and care needs. We probed for possible planning ‘triggers’–future events such as serious illness [10] that would kick-start planning–but even those were generally insufficient in practice to lead to the completion of a written plan. Avoidance of planning has been attributed by some to a reluctance to talk about death or dying [5,6]. In contrast, we found that interviewees were willing to talk about aspects of death, and particularly the practical and financial considerations involved in making funeral plans and wills. Interviewees were reluctant, however, to discuss the loss of physical or mental faculties, or any other deterioration that led to critical changes in social self; interviewees did not want to think about transitioning from being seen as a brother, wife, or mother, to being seen as a patient. Since these details may be important parts of a written ACP, it is possible that it is this–the confronting of the reality of life at the end of life, rather than the reality of death itself–that may form a key psychological barrier to deciding to create one’s own plan. Interviewees also acknowledged the unpredictability of end-of-life, which makes planning difficult, and potentially futile. It is worth noting too that some people are generally not good at formally planning ahead, or do not like to plan ahead, in all aspects of their lives [38].

Our findings indicate that the idea that family, friends, or health professionals could facilitate the ACP process are optimistic at best. Trust in and communication with family and friends has been identified as an important facilitator of advance care planning in some studies [8,21,34,38]. However, our findings echo those of Malcolmson and Brisbee [24] who found that a strong supportive family often constituted a reason *not to plan*, with people relying on the judgement of loved ones, rather than needing a written plan. Individuals were also reluctant to raise the topic with their own elderly relatives. Similarly, while trust in medical professionals has been identified as a facilitator of advance care planning [8,29,39], the possible enabling role of GPs had little support, and was generally seen as unrealistic given the lack of time for GP consultations, under-resourcing in the NHS, and lack of trust in GPs to make expert decisions in a specialist area.

These findings do not bode well for current policy of encouraging ACPs. It appears that current ACPs do not focus on what people are interested in planning for, and the areas they cover are picked up as and when needed by others. A simple framework such as the capability, opportunity, and motivation COM-B model [40] provides organising principles for behaviour change techniques. Thus, we have identified perceived lack of capability (‘I can’t predict my future care needs’), no clear opportunity (‘there’s plenty of time in the future, I’ll worry about it then’), and motivational deficits (‘I trust others, so there is no need for a written plan’). Whilst some authors, such as Cheung, Au, Ip et al. [41], propose that supportive interventions are required for ACP to be more effective, others argue that this will not reduce the effect of the factors undermining ACPs [3]. Our results suggest that the barriers are so fundamental that the policy itself needs a rethink, and we propose that ACP policy should focus on planning as an ongoing, conversational, process, rather than a document. This follows from Seymour et al’s [30] insight that the benefits of planning derive not from the plan itself, but from the process of communication required to produce it. This is also in line with Abel et al’s [42] proposal for a social model of care in advance care planning, as an alternative to the clinical model of death, which recognises the importance of a patient’s social network. A policy of promoting open communication between individuals and those (potentially) involved in their care, would therefore be more appropriatel than emphasising the need for a written plan.

Lastly, framing planning as facilitating ‘choice’, is potentially unhelpful, as the public are wise to the current limitations of choices in end-of-life care, due to the unpredictability of what might happen to them, limited resources in health and social care services, and personal, financial, and social support constraints. Equally as important, therefore, is building capacity in health and social care systems, and community support networks, to be able to respond to individuals’ wishes, whether planned or emergent, in real time as the need arises.

## Study limitations and conclusions

### Limitations

The qualitative stage of this study was completed before the Covid-19 pandemic began, whilst the quantitative stage was undertaken during the first year of the outbreak. Although our findings indicated minimal impact of the pandemic on views towards end-of-life care, future research would be valuable to explore any longer-term impact.

### Conclusion

This study brings insight into the older well’s knowledge of and attitudes towards ACPs. Although advance care planning is seen by individuals as a good idea in theory, there is considerable reticence in practice, with individuals reluctant to confront issues related to dying/loss of self. The study has exposed the inherent difficulties of asking people who are well to plan for an uncertain future, with most people concluding that written plans are something they cannot engage with ‘now’. Indeed, if the intention is to hand over control and choice to people about their care, then the choice exercised has been that of declining to participate. We propose that the policy for these people may need to shift from encouraging the older well to adopt written ACPs, to focusing on a flexible ‘real-time’ approach to care planning, centred around ongoing conversations between individuals and those involved in their care.

## Supporting information

S1 File Interview guide. (DOCX)

S2 File Survey questions. (DOCX)
