# Supplementary material for: Attitudes towards advance care planning amongst community-based older people in England
Source: PLoS One. 2024 Aug 21;19(8):e0306810. doi: 10.1371/journal.pone.0306810 (PMC11338439; doi:10.1371/journal.pone.0306810)
Supplement: S1 File — (DOCX) [file pone.0306810.s001.docx]

**S1. Interview guide**

**Section 1 - Awareness, knowledge and reaction to End of Life Care Planning**

1. I would like to hear your reactions to this…

*Show Generic Proposition Card and read out*

**Figure 1: Generic Proposition Card**

Start planning for your future care and support

None of us know how things will turn out as we get older. It’s quite possible that many of us will need caring for. Some of us might also lose mental capacity to make decisions ourselves, e.g. through dementia.

You can talk to your family and health care professionals (for example, your GP) about the sort of care you’d like if you become dependent or seriously ill.

It’s best to write down your plans so that those who care for you have a record.

Prompts:

Is there anything that you think is good about this idea (and why)?

Is there anything that is of concern to you about this idea (and why)?

Have you heard or thought about this idea before our research? If yes:

- Where did you hear or read about it?
- Who was proposing it and why?
- Have you discussed it? With whom?
- What are the implications for the person themselves; their family or the people close to them; the NHS and the medical people involved (GPs etc); the government.

Have you been involved in this type of activity? For whom?

Have you been involved it for yourself? If not, why not?

Who would you prefer to talk to about it and why? Who would you prefer not to talk to and why?

Ask only if previously mentioned any of the below:

Where did you hear about this? What was your reaction? Have you taken any action?

- Advance Care Planning (ACP)
- Advance Directive /Advance Statement (AD/AS)
- Advance Directive to Refuse Treatment (ADRT)
- Lasting Power of Attorney (LPA)

1. I would like to hear your reactions to this…

*Show Descriptor of Advance Care Plans (pilot and first main interview stage)/Example Advance Care Plan (final interview stage). Give the respondent a few minutes to read the statement.*

**Figure 2: Descriptor of Advance Care Plans**

It has been suggested that people should think in advance how they would like to be cared for if they become unable to care for themselves and less able to make decisions for themselves*.* Below are the sorts of decisions one needs to make in what is called an “Advance Care Plan”.

- -where you would like to be cared for (for example, in your home or in a care/nursing home)
- who you would wish to care for you if physically or mentally unable to care for yourself (family/friends or professional visiting carers or care/nursing home)
- treatments you would refuse.
- and who should make decisions for you if you are no longer able to do so

**Figure 3: Example Advance Care Plan**

This is a fictional statement based on examples provided by the Paul Sartori Foundation.

Alan Fawcett is healthy and is not anticipating any particular care needs. When he wrote this, he was hoping to provide information that would be useful in the event of an unpredictable event, such as an extensive stroke or head injury, which would impair his ability to make decisions.

He was offered the option of making his preferences legally binding by writing an Advance Decision to Refuse Treatment, but did not feel this was necessary.



**STATEMENT OF WISHES AND CARE PREFERENCES**

These are my hopes and wishes regarding my future care and treatment should I become unable to make those decisions for myself.

I am aware that this is not a legally binding document.

If I lose the capacity to make decisions, I wish for this document to be used byothers regarding my treatment and care.

My name:

Alan Fawcett

My date of birth:

24/3/1952

Name and address of my GP:

Dr Messer

Old Lane Surgery

A copy of this statement is with my GP: On completion a copy will be sent to my GP

Do you have an advance decision to refuse treatment? No


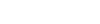
Who would you like to be involved in making decisions about your care if it ever becomes difficult for you to make decisions?

I would like the following to be consulted:

Robert Fawcett – my son

Daisy Fawcett – my daughter. Daisy is in the US and would need to be able to communicate partly by email ([Daisy101@live.co.uk](mailto:Daisy101@live.co.uk))

I give my permission for this

Do they have Lasting Power of Attorney? No

Do you have any special preferences or wishes regarding your future care?

I would like all efforts to be made to include me in decision-making. I prefer to have as much information as possible about my condition, even if that means hearing bad news.

If I were so physically or cognitively impaired as to be unable to make a decision, and if that was unlikely to change, I would not want any treatment aimed at extending life. I would want comfort measures only.

I have bad memories of asthma attacks as a child and have a particular fear of fighting for breath. I would particularly want treatment aims at relieving this distress, regardless of side effects.

Would you like your organs or tissues to be considered for donation?

Yes - I am a registered donor

If your condition deteriorates where would you most like to be cared for?

This would depend on how long I was likely to need care for. If the need was likely to be short term (eg: diagnosis of extensive cancer) I would like to be at home, but I would not like either of my children to give up work to make this happen. If the need was for specialised, 24 hour or long-term care, I would prefer to be in a care home.

Is there anything you would ideally like to avoid happening to you?

I would hate to have to use bed pans or incontinence pads. My preference would be to be catheterised if I become incontinent of urine. I am happy to accept the risks associated with this.

I would not want family members to provide very personal care.



Do you have any comments or wishes that you would like to share with others?

I have put together a document with some facts about my life and preferences for day to day living. I would like this to be available to anyone involved in my long-term care, if I am unable to speak for myself

Please write down the details of any family members or healthcare professionals who know about your wishes

Robert Fawcett – my son.

Daisy Fawcett – my daughter

Date written:

01/05 /2012

Your name and signature:

Alan Fawcett

Remember: you may wish to share a copy of this with your medical team e.g. your GP or nurse.

Prompts:

What do you think of these choices?

Are these likely to be realised and why (not)? Is there anything that limits choice?

What are the pros and cons of the preference to stay at home?

Are you aware there are attempts to encourage people to be cared for at home? Why do you think this is being suggested?

Who should advise on wishes and care preferences?

How would you feel if your GP or a hospital doctor suggested you might like to think about this? Would it affect your relationship with your doctor?

How would you broach this with your family?

When is it possible to make plans? When is it impossible?

Might someone change their mind? When?

**Section 2 - Thought provocation**

Here are some things people have said about care for those at the end of life. I am really interested in your reactions to each one I show you. This is about opinions, there are no absolute right answer or wrong ones. Please read both viewpoints and I would like your opinion on both viewpoints.

*Pass showcards to participants and read out. Rotate blocks A, B, C from interview to interview. Alternate starting with the ‘Some people think’ and ‘Other people think’ viewpoint between each interview, to balance any order effects within a couplet.*

**Figure 4: Viewpoint showcards**

*Block A - always start with 1 and rotate 2, 3, 4 from interview to interview*

1. Some people think telling your family your preferences for care is sufficient. Others think it should be in writing, witnessed and held by a third party such as the NHS.
2. Some people want the GP to make the call that the time is right to create an Advance Care Plan. Others think you should wait for a specific trigger such as a serious illness.
3. Some people are very happy to take a doctor's advice on planning for care. Others want to take charge of their own care.
4. Some people think Advance Care Plans must be followed to the letter. Others think it should be an expression of wishes but no more than that.

*Block B – rotate 5 and 6*

1. Some people think the family should step in and help the person to live at home. Others think the state should make provisions for this to happen
2. Some of those needing care say they don’t want to be a burden to their family. But others say they secretly hope and expect their family to step in.

*Block C – rotate 7, 8, 9*

1. Some people think it is good everyone has choices for end of life care. Others think that the only people that have real choices are those that can afford them
2. Some people find it difficult to write an Advance Care Plan because they do not like thinking about losing their health or their independence. Others just think that a written plan is not appropriate.
3. Some people say staying at home risks you not getting reliable or round the clock professional care and pain relief. Others think they could live this rather than go into a hospital.

Prompts:

Which way do you lean and why?

What qualifications would you make (e.g. I agree with X view if.... }?

What questions does it raise for you?
