# Supplementary material for: Attitudes towards advance care planning amongst community-based older people in England
Source: PLoS One. 2024 Aug 21;19(8):e0306810. doi: 10.1371/journal.pone.0306810 (PMC11338439; doi:10.1371/journal.pone.0306810)
Supplement: S2 File — (DOCX) [file pone.0306810.s002.docx]

**S2. Survey questions**

**Advance Care Planning Survey**

This survey is about health and care.

**Q1a.** Which of these are you aware of?

Funeral plans

Medical travel insurance

Eye or hearing tests

Making a will

Home equity release

Advance Care Planning

…………………………………………………………………………………….

Lasting Power of Attorney for property and financial affairs

Lasting Power of Attorney for health and welfare

………………………………………………………………………………………

Private medical insurance

Adapting my home for old age

…………………………………………………………………………………….

None of these

*(Randomised order, excluding “none of these”, which always comes last; two LPAs kept together)*

*List reduced to those items aware of at Q1a*

**Q1b.** Which of these, if any, have you looked into, either for yourself or for someone else?

**Q1c.** Which, if any, have you completed for yourself?

**Q1d.** Which, if any, have you helped someone else with?

**Q1e.** Which, if any, of these could you explain fully to someone else without help or without checking beforehand.

***IF AWARE OF Advance Care Planning at Q1a***

**Q2** Please explain what, if anything, you know about Advance Care Planning in your own words.

**Q2a.** When should someone do an Advance Care Plan?

Don’t know

Can’t remember

Type here……………………………………………………………………………………………

**Q2b**. What should someone state they want in an Advance Care Plan?

Don’t know

Can’t remember

Type here …………………………………………………………………………………………….

***ASK ALL***

**Q3** It has been suggested that people should think in advance how they would like to be cared if they become unable to care for themselves and less able to make decisions for themselves*.* Below are the sorts of decisions one needs to make in an “Advance Care Plan”.

*(KEEPING THE BELOW IN VIEW FOR Q3a)*

-where you would like to be cared for (for example, in your home or in a care/nursing home)

- who you would wish to care for you if physically or mentally unable to care for yourself (family/friends or professional visiting carers or care/nursing home)

- treatments you would refuse.

- and who should make decisions for you if you are no longer able to do so.

**Q3a**. Have you written an Advance Care Plan for yourself covering these issues or told anyone about your wishes if you were to need care? Which one statement best describes you? *Select one answer only.*

1. I haven’t thought about what my wishes are
2. I know what my wishes are, but I just haven’t told anyone or written them down
3. I have told someone my wishes, but haven’t put these in writing
4. I have put my wishes in writing
5. Not sure/ can’t remember

**Q3b.** What prompted you or would prompt you to think about an Advance Care Plan for yourself? *Click all that apply.*

*(Randomised order, except for “other” and “can’t remember” which come last)*

A serious illness/ condition affecting me

A serious illness/ condition affecting someone else close to me

A pandemic like Covid 19

A GP suggesting it

A hospital consultant suggesting it

A nurse suggesting it

A carer suggesting it

A friend/relative suggesting it

Learning about it in the media (TV/radio/press/social media)

Discovering it on the internet

Concerned that my family would not know what type of care I would want

It could help me to stay at home rather than go into care

It could help me get the type of care I want

It would help doctors and nurses to make the right decisions for me

My family would know what I want in terms of care

Other (write in)

Don’t know/ Can’t remember

*If (a) or b) or (c) … or (e) at Q3a.*

**Q3c.** Are any of these the reasons why you did not/would not write an Advance Care Plan for yourself now? *Click all that apply.*

I can’t predict my future health, finances, who might be able to help me

I don’t want to think about it yet

I don’t need to think about it yet

I don’t want to do one in case my circumstances change

No need as my family/friends would know my wishes

No need as I trust my family/friends to step in

No need as Social Services and the NHS would look after me

I have other more important or pressing issues in my life

Don’t know to whom to give a written Advance Care Plan

An Advance care Plan could get lost

An Advance care Plan could get ignored

Don’t know whom to trust with my Advance Care Plan

Forgot to do anything about it

I don’t know how to write an Advance Care Plan

I don’t think that having an Advance Care Plan will make any difference to the care I receive.

Writing an Advance Care Plan would upset me

Writing an Advance Care Plan would upset any family/friends that I involved in the process

Setting up a Lasting Power of Attorney for Health and welfare might be better

………………………………………………………………………………………………….

Other (write in)

Don’t know

*(Randomised order, except for ”other” and “don’t know”, which come last)*

ASK ALL

**Q4** In an earlier stage of research members of the public gave different ways in which they reacted to the idea of doing an Advance Care Plan. Here are some of the things individuals said. How strongly do you personally disagree or agree with each one?

*(5 point Strongly disagree – Strongly agree, randomised order of sets of scales)*

Think there is no need to do an Advance Care Plan until a serious illness or disability occurs

Think there is no need to do an Advance Care Plan until the specific needs are known

Be more inclined to write a will as something positive to do now

Be more inclined to plan my funeral as something positive to do now

Find it too depressing or upsetting to think about now

Prefer to appoint someone with a Lasting Power of Attorney for my health and welfare

Suspect the motives of whoever is trying to encourage me to do an Advance Care Plan

Look for some valid reasons for not doing an Advance Care Plans

It seems so remote so make a joke about it

Be irritated or angry at this unnecessary intrusion in one’s life

Be fatalistic – whatever will be, will be

Wait as there will probably be better solutions by the time care is needed

Be more inclined to simply tell their family/friends one’s wishes rather than producing a written Advance Care Plan.

Palliative care is so good now there is little chance of suffering pain

The Covid 19 pandemic made me think more about care for elderly people I know

The Covid 19 pandemic made me think more about my own care should the need arise

ASK ALL

**Q5a.** Would you prefer to tell someone your care wishes or put it in writing?

Tell someone

Put it in writing

Neither – no intention of doing an Advance Care Plan

**Q5b.** If you told someone your wishes or wrote an Advance Care Plan who would you trust to be involved in helping you to do this?

My partner

Another relative

Close friend

GP

Your hospital consultant

Care home/nursing home /assisted living manager

Solicitor

Other (write in)

**Q5c.** *If in writing at Q5a.*

Who would you entrust to keep the written Advance Care Plan?

*(List as above)*

**Q6.** Which of these statements apply to you currently about your family? *Click all that apply.*

I have family (this includes husband, wife, partner) and we live in the same house

I have family living nearby

I have family living some distance away in the UK

I have family living abroad

None of these / I have no family

ASK ALL

**Q7.** How is your health in general?

Very good

Good

Fair

Bad

Very bad

**Q8.** Which one of these, best describes your home?

My own private home (owned/rented)

Living in a relative’s home

Assisted living/sheltered home (with a manager or warden)

Care home

Nursing home

Other {open}

**Q9.** Do you have [elderly] relatives (aged 65+) [that you may feel responsible for], that you may feel you want to discuss care matters with] [who live in England]?

Yes, parents

Yes, aunts/uncles

Yes brothers/sisters

Yes other relatives

No

END
